# Using Femtosecond Laser to Create Customized Corneal Flaps for Patients with Low and Moderate Refractive Error Differing in Corneal Thickness

**DOI:** 10.1371/journal.pone.0121291

**Published:** 2015-03-25

**Authors:** Chi Zhang, Jingbin Che, Jianhong Yu, Linli Yu, Dan Yu, Gangping Zhao

**Affiliations:** 1 Department of Ophthalmology, The First People’s Hospital of Foshan, Foshan, Guangdong, China; 2 Department of Ophthalmology, People’s Hospital of Laiwu, Laiwu, Shandong, China; Bascom Palmer Eye Institute, University of Miami School of Medicine;, UNITED STATES

## Abstract

This study is designed to evaluate the visual outcomes, accuracy, and predictability of corneal flaps with different thicknesses created by 60-kHz femtosecond laser according to different corneal thicknesses in the patients with low and moderate refractive error. A total of 182 eyes were divided according to the central corneal thickness (470μm–499 μm in Group A, 500μm–549 μm in Group B, and 550μm–599 μm in Group C) and underwent femtosecond laser-assisted LASIK for a target corneal flap thickness (100 μm for Group A, 110 μm for Group B, and 120 μm for Group C). Uncorrected distance visual acuity (UDVA), corrected distance visual acuity (CDVA), and refractive status were examined. The flap thickness of each eye was measured by anterior segment optical coherence tomography (AS-OCT) on 30 points at 1-month follow-up to assess the accuracy and predictability. Postoperatively, at least 75% of eyes had a UDVA of 20/16 or better, less than 2% of eyes lost one line, over 30% of eyes gained one or more lines in CDVA, at least 95% of eyes had astigmatism of less than 0.25 D, all eyes achieved a correction within ±1.00 D from the target spherical equivalent refraction. The visual and refractive outcomes did not differ significantly in all groups (P >0.05). The mean flap thickness was 100.36± 4.32 μm (range: 95–113 μm) in Group A, 111.64 ± 3.62 μm (range: 108–125 μm) in Group B, and 122.32 ± 2.88 μm (range: 112–128 μm) in Group C. The difference at each measured point among the three groups was significant (P < 0.05). The accuracy and predictability were satisfactory in all three groups. In conclusion, this customized treatment yielded satisfactory clinical outcomes with accurate and predictable flap thickness for patients with low and moderate refractive error.

## Introduction

Laser in situ keratomileusis (LASIK) has been the most commonly performed refractive surgical procedure. A critical step in this procedure is to create the corneal flap. Femtosecond laser technology has emerged as an alternative way to precisely create the epithelial-stromal flap in LASIK(FS-LASIK), which is crucial for obtaining an appropriate residual stromal thickness and achieving satisfactory visual and refractive outcomes[1–4].

Previous studies have reported the flap-producing characteristics of LASIK with different femtosecond laser technologies and their relationships to visual and refractive outcomes[5–10]. Different femtosecond laser energies [5,6], different femtosecond laser systems[7], corneal flaps with different diameters and cutting edge [8], and corneal flaps with different thicknesses made by femtosecond laser [9,10] were compared. The patients in those studies, however, were usually grouped by their refractive statuses or corneal flap cutting patterns. Making customized corneal flaps for patients with different corneal thicknesses has not been investigated yet.

In the present study, 60-kHz IntraLase femtosecond laser was used to create customized corneal flaps according to individual corneal thickness in the patients with low and moderate refractive error. Postoperative visual and refractive outcomes were analyzed to explore the feasibility and merits of this new customized treatment.

## Materials and Methods

### Subjects

The patients who underwent bilateral femtosecond laser-assisted LASIK to correct myopia or myopic astigmatism between October 2011 and January 2012 at Affiliated Foshan Hospital of Sun Yat-sen University were enrolled in this study. The following enrollment criteria were used: preoperative spherical equivalent refraction between -1.00 diopter (D) and -5.00 D; preoperative cylindrical equivalent refraction between -0.25D and -1.50 D; preoperative corneal curvature between 39.8D and 46.2D. All subjects provided written consent to participate in this study. All procedures were approved by the Ethics Committee of The First People’s Hospital of Foshan in compliance with the tenets of the Declaration of Helsinki and obtained ethics.

The patients were divided into three groups according to their central corneal thickness: 470μm-499 μm for Group A, 500μm-549 μm for Group B, and 550μm-599 μm for Group C. The flap thickness was set as 100 μm for Group A, 110 μm for Group B, and 120 μm for Group C.

Preoperative assessment included uncorrected distance visual acuity (UDVA), corrected distance visual acuity (CDVA), intraocular pressure (IOP), tear break-up time (TBUT), Schirmer test, corneal topography (HUM PHREY HCT993, Orbscan; Bausch & Lomb Inc, Rochester, NY, USA), keratography (TOPCON OM-4, Japan), axial length (Ocuscan Rxp, Carl Zeiss Meditec AG), dilated fundoscopy, slit-lamp microscopy, and anterior segment optical coherence tomography (AS-OCT, anterior segment OCT 2000, Topcon, Japan). All visual acuity measurements were performed using Snellen charts.

Standard postoperative treatment consisted of tobramycin—dexamethasone (TobraDex) eyedrops every 2 hours for the first 24 hours after operation and every 6 hours for the following 6 days. Artificial tear eyedrops were applied as required for 1 month. Follow-up examinations consisted of refraction status, UDVA, CDVA, and slit-lamp microscopy at 1 week and 1 month, AS-OCT examination for flap thickness at 1 month after operation.

### FS-LASIK Procedure

All surgeries were performed by the same experienced surgeon (Gangping Zhao) under topical anesthesia. The 60-kHz IntraLaser femtosecond laser system (IntraLase Corp, Irvine, CA, USA) was used to create the corneal flap. The settings were a diameter of 8.5–8.8 mm, a side cutting energy of 0.70 μJ, a side cutting angle of 70°, a raster pattern energy of 0.75 μJ, an interval between raster line and laser spot of 7 μm, and a hinge angle of 45°, with the superior hinged flap. After creating the flap, ablation of the stromal was performed with the VISX Star S4 excimer laser (VISX Inc, Santa Clare, CA, USA) in a routine manner. All eyes were targeted for emmetropia.

### Flap Thickness Measurement

**Fig 1:**
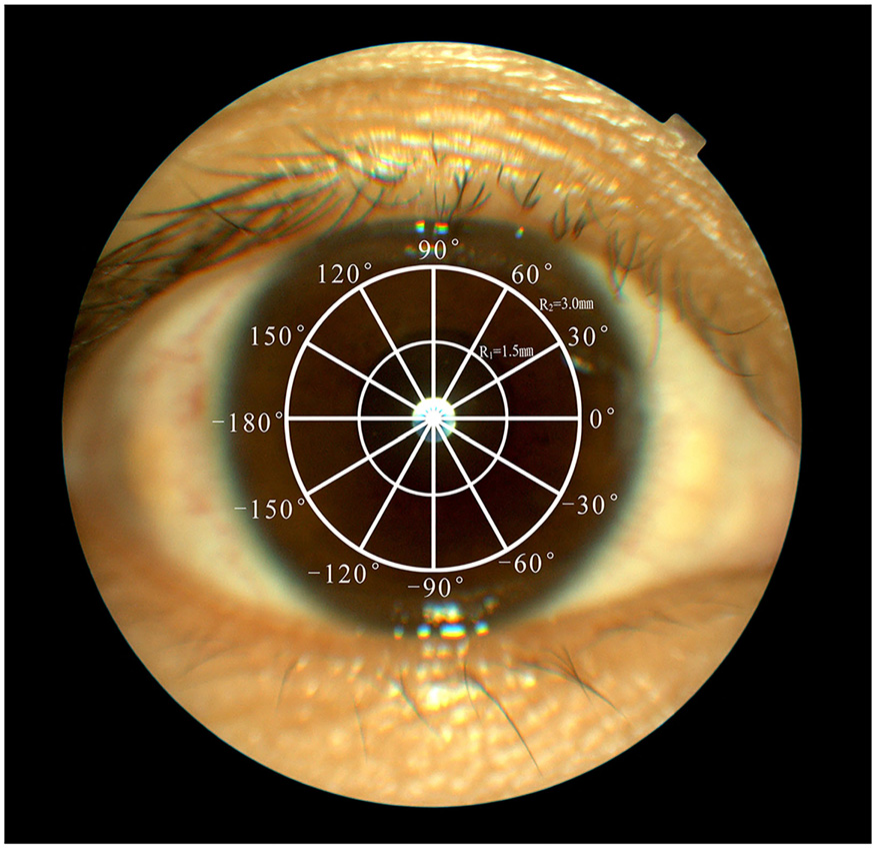
Optical coherence tomography scan pattern. Six meridians (0°, 30°, 60°, 90°, 120°, and 150°) were selected to measure the thickness of the whole cornea flap. The five measurement points on each meridian were ±0.5 mm, ±1.5 mm, and ±3.0 mm from the center. “+” denotes superior side, and “-” denotes inferior side.

We measured flap thickness using an AS-OCT and the method was similar with the reported study [11]. The acquired images had a resolution of 884 × 512 pixels where a single pixel covered the area of 5 × 10 μm at the colour resolution of 8 bits/pixel. All high-resolution images were acquired by the same skilled examiner Jingbing Che at 1 month after surgery and stored in the database for later retrieval and analysis. Six meridians, 0° to -180°, 30° to -150°, 60° to- 120°, 90° to -90°, 120° to -60°, and 150° to -30°, were selected for measurements. Each meridian scan had 5 measuring points: 1 point was at the central zone (±0.5 mm from the flap vertex), 2 points at the paracentral zone (±1.5 mm from the flap vertex), and 2 points at the peripheral zone (±3 mm from the flap vertex). Therefore, a total of 5 locations per meridian and a total of 30 locations per flap were obtained for analysis (Fig. 1).

### Statistical Analysis

Statistical analyses of clinical outcomes were performed by using SPSS version 15.0 (SPSS Inc, Chicago, IL, USA) software. Analysis of variance (ANOVA) and adjusted Bofferoni test were used to compare the results of the three groups. A *P* value less than 0.05 was considered statistically significant.

## Results

### Preoperative Characteristics

A total of 91 patients (182 eyes) were enrolled in this study: 30 in Group A, 30 in Group B, and 31 in Group C. The preoperative clinical characteristics of all patients are shown in Table 1.

**Table 1:** Preoperative characteristics of the three groups of patients with refractive error.

Parameter	Group A	Group B	Group C	P Value
Patients (n)	30	30	31	
Eyes (n)	60	60	62	
Sex [n (%)]				
Female	14(46.7)	17(56.7)	13(42.0)	0.36
Male	16(53.3)	13(46.7)	18(58.0)	
Age (years)	29.81±5.15	28.33±4.69	26.77±4.26	0.67
IOP (mmHg)	13.5±1.6	13.9±1.1	13.2±1.2	0.53
CCT (μm)	491.95±9.21	531.62±11.18	576.83±16.10	0.03
K value	44.05±1.74	43.61±1.27	44.26±1.08	0.46
Schirmer test [n (%)]				
≤5 mm	4(6.7)	6(10.0)	3(4.8)	0.58
6–10 mm	12(20.0)	12(20.0)	14(22.6)	
≥10 mm	44(73.3)	42(70.0)	45(72.6)	

IOP, intraocular pressure; CCT, central corneal thickness. All data of IOP, CCT, and K value are presented as mean ± standard deviation (SD) of all eyes in relevant groups.

### Postoperative Visual Acuity

**Fig 2:**
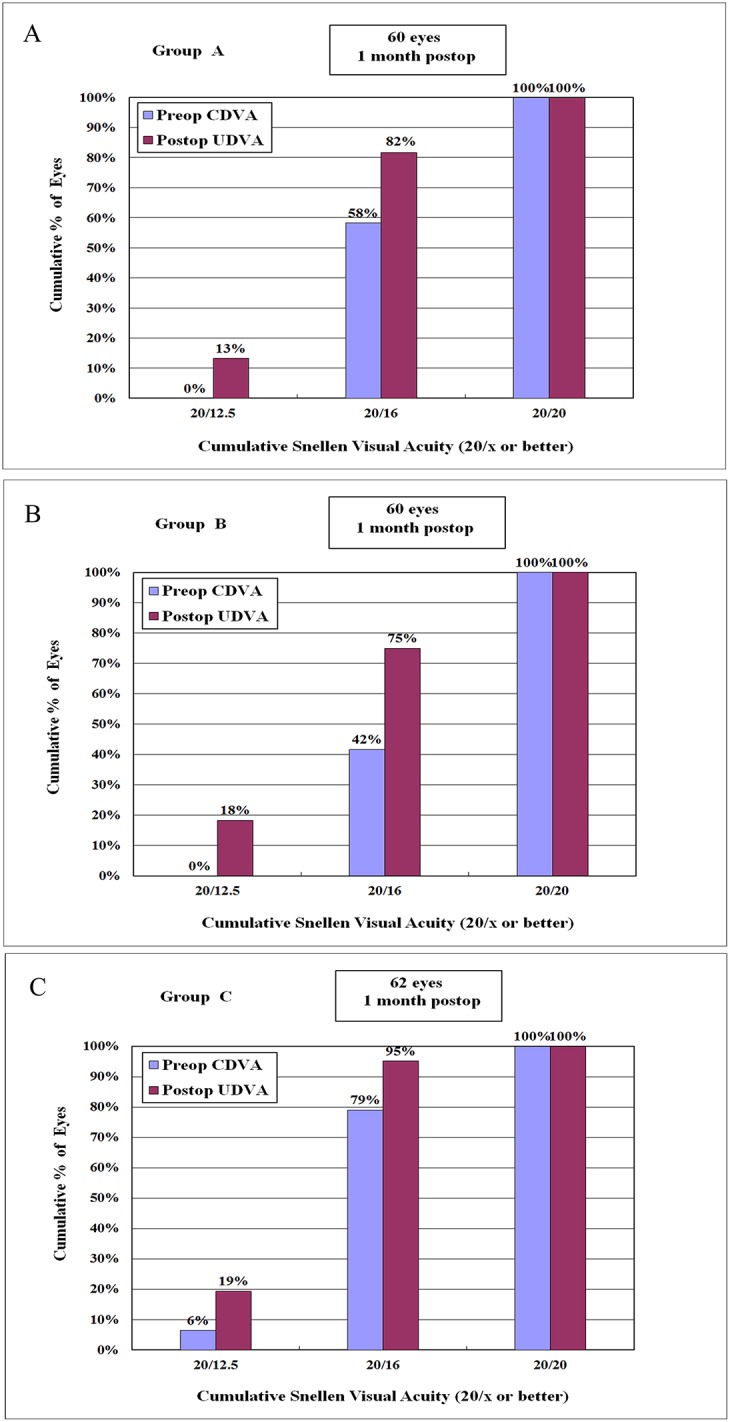
Preoperative corrected distance visual acuity (CDVA) and 1-month postoperative uncorrected distance visual acuity (UDVA) for Group A (A), Group B (B), and Group C (C). The central corneal thickness was 470–499 μm in Group A, 500–549 μm in Group B, and 550–599 μm in Group C; the target corneal flap thickness was 100 μm for Group A, 110 μm for Group B, and 120 μm for Group C. Preoperative CDVA was 20/20 or better, and 1-month postoperative UCVA was 20/20 or better for all the groups.

Both preoperative CDVA and postoperative UDVA ranged from 20/20 to 20/12.5 in the three groups. The postoperative UDVA was 20/16 or better in 75%–95% of eyes and was 20/20 or better in all eyes (Fig. 2).

**Fig 3:**
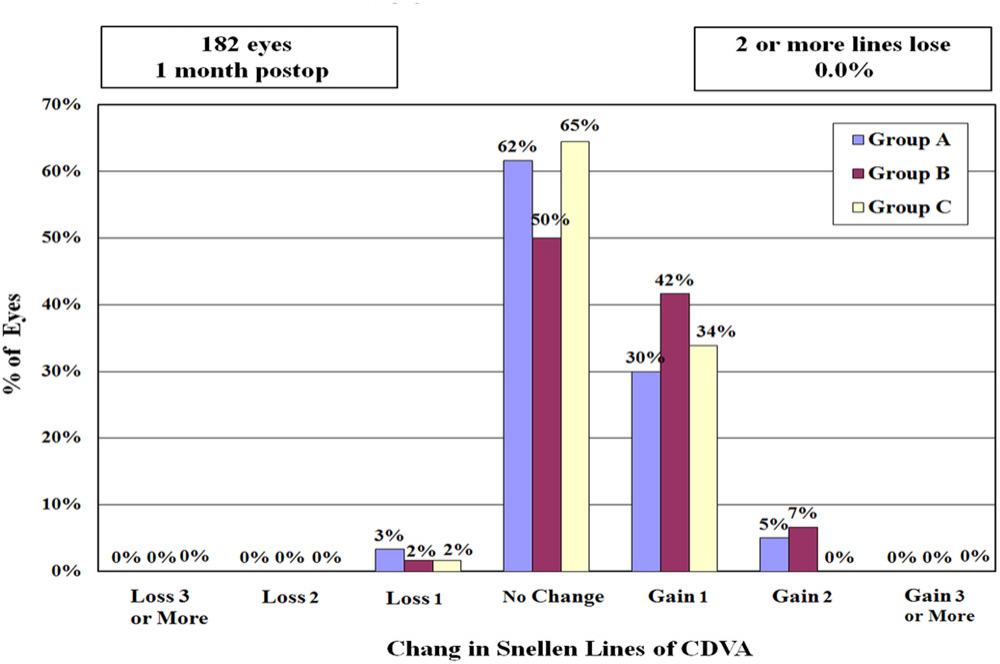
Changes in Snellen CDVA for the three groups at 1 month after operation. The percentage of eyes lost 1 line of preoperative CDVA ranges from 1% to 2%, and the percentage of eyes gained 1 or more lines ranges from 34% to 49% for the three groups.

The percentages of eyes gained 1 or more lines of CDVA ranged from 34% to 49% in the three groups; the percentages of eyes with no change ranged from 50% to 65%; the percentages of eyes lost 1 line ranged from 1% to 2% (all P > 0.05) (Fig. 3).

### Refractive Status

**Fig 4:**
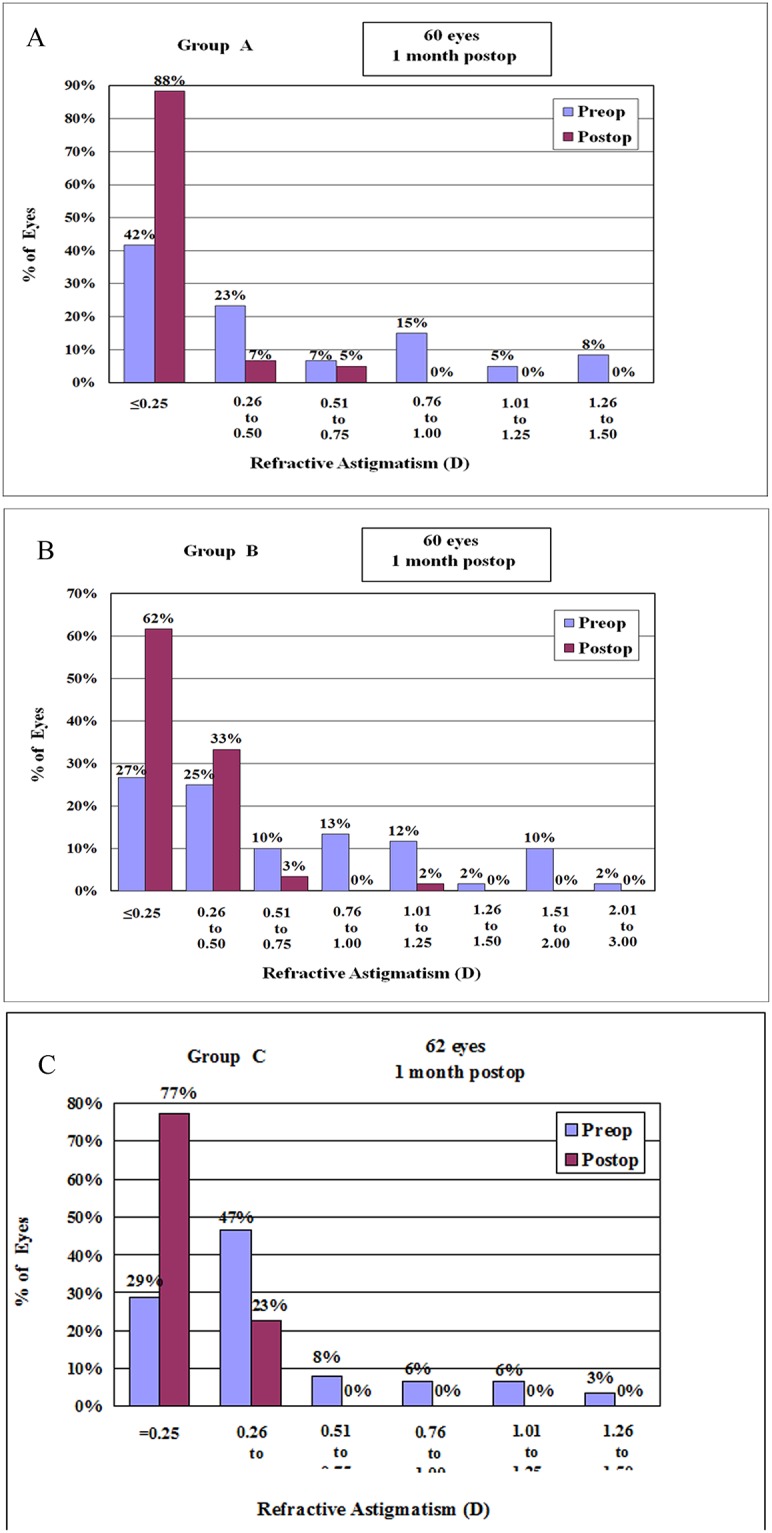
The 1-month postoperative refractive astigmatism for Group A (A), Group B (B), and Group C (C). The percentage of eyes with refractive astigmatism less than 0.50 D ranges from 95% to 100%. Refractive astigmatism between 1.01 D and 1.25 D was observed only in 2% of the eyes in Group B.

Postoperative astigmatism was less than 0.75 D in 98%–100% of eyes and less than 0.25 D in at least 62% of eyes; it ranged from 1.01 D to 1.25 D in only 2% of eyes in Group B (Fig. 4).

**Fig 5:**
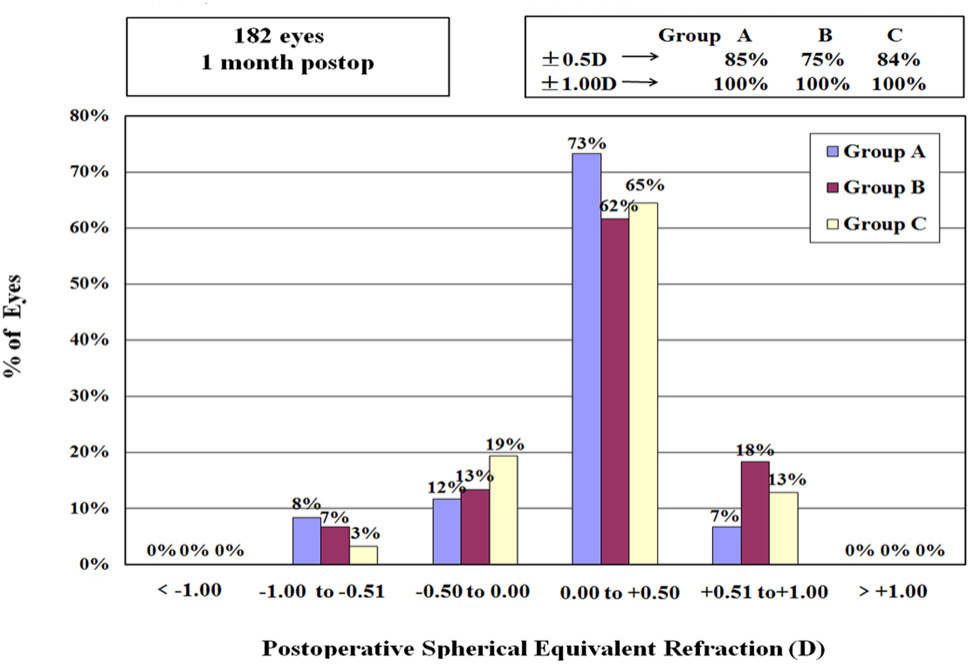
Postoperative spherical equivalent refraction for the three groups at 1 month after operation. The percentage of eyes with spherical equivalent refraction within ±0.50 D ranges from 75% to 85%. In all eyes, spherical equivalent refraction was within ±1.00 D.

**Fig 6:**
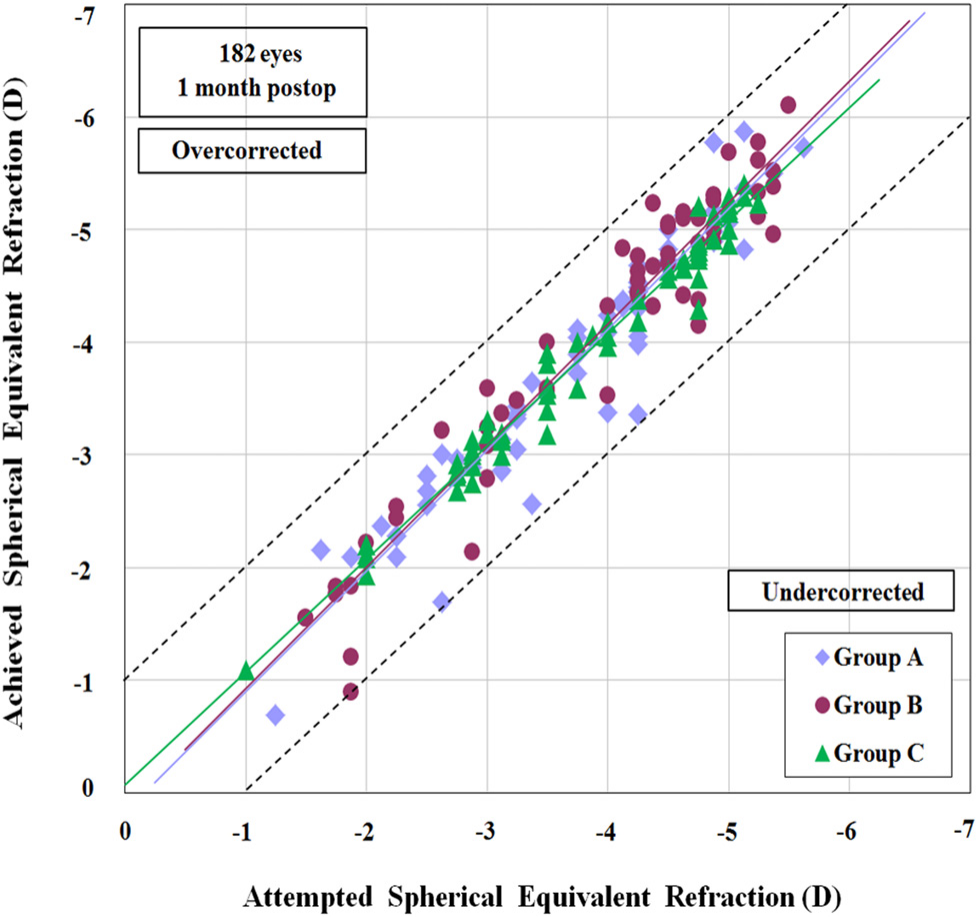
Scattergram of the attempted and 1-month postoperative achieved spherical equivalent refraction for Group A (purple), Group B (crimson), and Group C (green). The two peripheral dotted lines represent 1.00 D of undercorrection and overcorrection. All eyes achieved a correction within ±1.00 D from the target spherical equivalent refraction.

In all groups, the postoperative spherical equivalent refraction was within ±0.50 D of the target refraction in at least 75% of eyes and within ±1.00 D in all eyes (Fig. 5 and Fig. 6).

**Fig 7:**
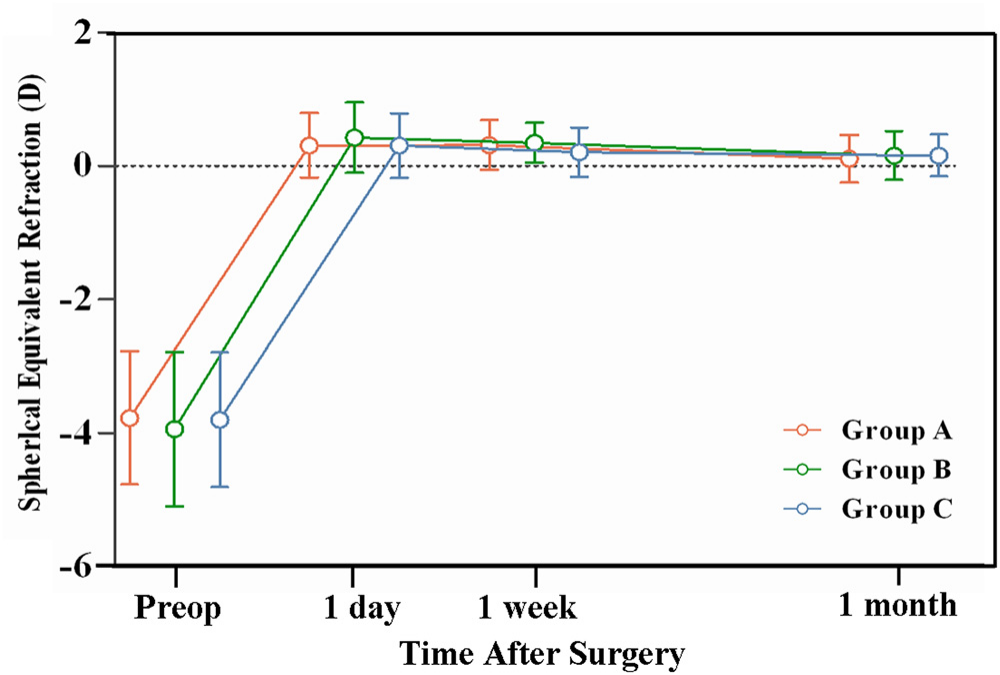
Stability of postoperative spherical equivalent refraction for Group A (red), Group B (green), and Group C (blue). A change of more than ±0.50 D was found in only 1.6%–9.3% of eyes in the three groups.

The postoperative refractive predictability results of the three groups were not significantly (P > 0.05). The changes more than ±0.50 D of the postoperative spherical equivalent refraction between 1 week and 1 month after surgery were 9.3% in Group A, 5.5% in Group B and 1.6% in Group C (Fig. 7).

### Mean Flap Thickness

The mean corneal flap thickness, as calculated by averaging the 30 points obtained from each eye, was 100.36 ± 4.32 μm (range: 95–113 μm) in Group A, 111.64 ± 3.62 μm (range: 108–125 μm) in Group B, and 122.32 ± 2.88 μm (range: 112–128 μm) in Group C (P <0.05). In all groups, the corneal flap thickness was the lowest at the central point (Table 2), but no significant differences were found among the measured points in any group (P >0.05).

**Table 2:** Flap thickness measured in each group at 30 points.

Measurement Points	Flap Thickness (μm)				
	- Peripheral	- Paracentral	Central	+ Paracentral	+ Peripheral
Group A					
0° meridian	100.95±4.21	100.61±4.39	98.58±3.12	100.58±4.19	101.00±4.65
30° meridian	100.80±4.53	100.60±4.16	98.82±3.29	100.67±4.41	101.30±5.02
60° meridian	101.00±4.82	100.81±4.17	98.87±3.37	100.73±4.41	101.07±4.60
90° meridian	101.15±4.97	100.77±3.97	98.87±3.37	100.88±4.49	101.13±4.67
120° meridian	101.05±4.87	100.69±4.10	98.78±3.03	100.72±4.11	101.53±5.20
150° meridian	100.85±4.30	100.78±4.22	98.93±2.96	100.80±4.46	101.28±4.68
Group B					
0° meridian	112.00±3.94	111.50±3.20	110.30±2.49	111.85±3.71	112.35±4.48
30° meridian	112.10±3.92	111.35±3.24	110.30±2.14	111.90±4.00	112.15±3.84
60° meridian	112.00±3.94	111.40±3.38	110.13±2.19	111.90±4.00	112.35±3.64
90° meridian	112.35±3.99	111.35±3.27	110.00±1.94	111.80±3.91	112.15±3.82
120° meridian	112.55±4.15	111.65±3.57	110.35±2.48	111.85±3.84	112.40±3.85
150° meridian	112.40±4.37	111.70±3.51	110.45±2.52	111.70±3.79	112.75±3.98
Group C					
0° meridian	122.92±2.81	121.87±2.83	120.82±2.62	122.26±2.70	123.24±2.74
30° meridian	122.85±2.83	121.97±2.72	120.50±3.28	122.32±2.64	123.24±2.74
60° meridian	122.95±2.58	121.92±2.73	120.45±3.31	122.47±2.80	123.18±2.73
90° meridian	123.05±2.61	122.10±2.71	121.02±2.62	122.59±2.77	123.24±2.74
120° meridian	123.10±2.66	122.26±2.70	121.06±2.77	122.73±2.85	123.18±2.73
150° meridian	123.09±2.66	122.26±2.70	121.06±2.77	122.82±2.83	123.18±2.73

Notes. “+” denotes superior side and “-” denotes inferior side. All data are presented as mean ± SD of all eyes in relevant groups.

### Flap Uniformity

Table 3 and Table 4 show the mean differences between the target and achieved flap thickness at each measurement point.

**Table 3:** Deviations from the target flap thickness of each group measured at 30 points.

Measurement Points	Deviation from Target Flap Thickness (μm)				
	- Peripheral	- Paracentral	Central	+ Paracentral	+ Peripheral
Group A					
0° meridian	1.09±4.20	0.60±4.38	-1.42±3.12	0.58±4.19	1.13±4.65
30° meridian	1.06±4.53	0.63±4.16	-1.18±3.29	0.67±4.41	1.30±5.02
60° meridian	1.10±4.82	0.74±4.17	-1.13±3.37	0.73±4.41	1.06±4.59
90° meridian	1.15±4.97	0.73±3.97	-1.13±3.37	0.88±4.49	1.13±4.69
120° meridian	1.05±4.87	0.68±4.38	-1.22±3.03	0.72±4.11	1.28±5.21
150° meridian	1.01±4.29	0.75±4.22	-1.07±2.96	0.80±4.46	1.18±4.68
Group B					
0° meridian	2.00±3.94	1.50±3.19	0.30±2.12	1.85±3.71	2.35±4.48
30° meridian	2.10±3.92	1.35±3.24	0.30±2.14	1.90±4.00	2.15±3.84
60° meridian	2.10±4.10	1.40±3.38	0.15±2.19	1.90±4.01	2.35±3.64
90° meridian	2.35±3.98	1.35±3.27	0.20±1.94	1.80±3.91	2.15±3.82
120° meridian	2.55±4.15	1.65±3.57	0.35±2.48	1.85±3.84	2.40±3.85
150° meridian	2.40±4.37	1.70±3.51	0.45±2.52	1.70±3.79	2.75±3.98
Group C					
0° meridian	2.92±2.81	1.87±2.83	0.82±2.62	2.26±2.79	3.24±2.71
30° meridian	2.85±2.83	1.97±2.72	0.90±3.28	2.32±2.64	3.24±2.75
60° meridian	2.95±2.58	1.91±2.73	0.85±3.31	2.47±2.80	3.18±2.73
90° meridian	3.05±2.61	2.09±2.71	1.03±2.62	2.58±2.77	3.24±2.74
120° meridian	2.92±2.81	1.87±2.83	0.82±2.62	2.26±3.79	3.24±2.74
150° meridian	3.09±2.66	2.26±2.70	1.06±2.77	2.82±2.83	3.18±2.73

Notes. “+” denotes superior side and “-” denotes inferior side. All data are presented as mean ± SD of all eyes in relevant groups.

**Table 4:** Accuracy of achieved flap thickness in each group measured at 30 points.

Measurement Points	Coefficient of Variance				
	- Peripheral	- Paracentral	Central	+ Paracentral	+ Peripheral
Group A					
0° meridian	0.04	0.04	0.03	0.04	0.04
30° meridian	0.05	0.04	0.03	0.04	0.05
60° meridian	0.05	0.04	0.03	0.04	0.05
90° meridian	0.05	0.04	0.03	0.04	0.05
120° meridian	0.05	0.04	0.03	0.04	0.05
150° meridian	0.04	0.04	0.03	0.04	0.05
Group B					
0° meridian	0.04	0.03	0.03	0.03	0.04
30° meridian	0.03	0.03	0.02	0.04	0.03
60° meridian	0.04	0.03	0.02	0.04	0.03
90° meridian	0.04	0.03	0.02	0.03	0.03
120° meridian	0.04	0.03	0.02	0.03	0.03
150° meridian	0.04	0.03	0.02	0.04	0.04
Group C					
0° meridian	0.02	0.02	0.02	0.02	0.02
30° meridian	0.02	0.02	0.02	0.02	0.02
60° meridian	0.02	0.02	0.02	0.02	0.02
90° meridian	0.02	0.02	0.02	0.02	0.02
120° meridian	0.02	0.02	0.02	0.02	0.02
150° meridian	0.02	0.02	0.02	0.02	0.02

Notes. “+” denotes superior side and “-” denotes inferior side.

**Fig 8:**
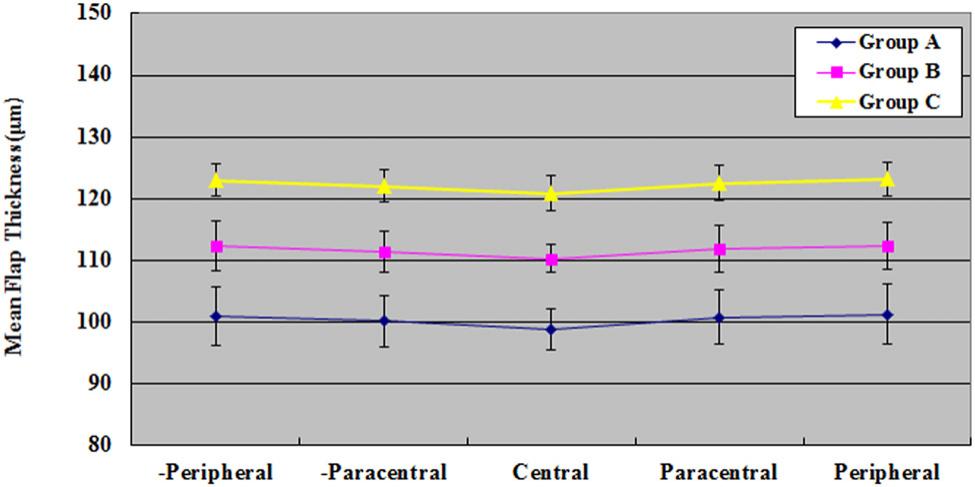
Comparison of the 1-month postoperative central, paracentral, and peripheral mean flap thicknesses among the three groups. “+” denotes superior side, and “-” denotes inferior side.

The max deviations from the target thickness at 30 points were 5 μm in Group A, 10 μm in Group B, and 8 μm in Group C. The average thickness of the corneal flaps at central, paracentral, and peripheral points was even in each group (Fig. 8) (P >0.05), implying the high regularity. The deviation of corneal flap thickness was increased from the central points to the peripheral points in all groups and was higher in Group C than in Group B and Group A. The accuracy of achieved flap thickness, as indicated by the coefficient of variance, was higher in Group C than in other two groups.

### Complications

No serious complications, such as flap tears, corneal ectasia, buttonholes, and incomplete passes, were observed in all the three groups during this study. Suction loss occurred in one eye in Group A and two eyes in Group C during the treatments. Bubbles of gas in the anterior chamber were seen in 5 eyes in Group A, 3 eyes in Group B, and 6 eyes in Group C. Subconjunctival hemorrhage was found in 5 eyes in Group A, 6 eyes in Group B, and 4 eyes in Group C. No eyes experienced delayed visual recovery or visual loss, and no flap folds or haze were seen during follow-up.

## Discussion

In the current study, we used femtosecond laser technology in LASIK to create customized corneal flaps according to different corneal thicknesses for three groups of patients with refractive error. The postoperative clinical outcomes of the three groups were satisfactory. All patients achieved good postoperative visual acuity and refraction. The corneal flaps were planar and predictable.

In terms of the efficacy of the customized treatment, postoperative UDVA of all the eyes were 20/20 or better, and at least 75% of eyes were better than 20/16. The astigmatism was below 0.25 D in more than 62% of eyes, and was between 1.01 D and 1.25 D in only 2% of eyes in Group B. At least 75% of eyes had postoperative spherical equivalent refraction within ±0.50 D of the target refraction. Less than 2% of eyes lost 1 line, whereas over 30% of eyes gained 1 or more lines in CDVA, indicating the safety of this treatment. All eyes achieved a correction within ±1.00 D from the target spherical equivalent refraction, indicating the predictability. The findings are similar to those observed by Prakash et al. [12] who used the same platform. However, the change of postoperative spherical equivalent refraction was seen in 9.3% (Group A), 5.5% (Group B), and 1.6% (Group C) of eyes in our study as compared to 6.7% (at 100-μm flap thickness), 3.3% (110-μm), and 5.0% (120-μm) of eyes in their research, respectively. This discrepancy in stability may be explained by that our patients received the examination at 1 week and 1 month after operation, whereas the patients in Prakash’s study received the examination at 2 weeks and 1 month after operation.

Femtosecond laser-assisted flap creation in LASIK is associated with the patients’ postoperative outcome closely. The measurement and analysis of the uniformity and accuracy of flap thickness has become very significant and meaningful. AS-OCT is a fast, noninvasive, noncontact procedure that can provide high resolution, cross-sectional images of the cornea at any appointed meridian. Especially, for central flap thickness measurement, AS-OCT is also superior to ultrasound pachymetry and Orbscan [13, 14]. Nowadays, AS-OCT has been widely used to measure the thickness of the whole corneal flap [15–17]. However, the resolution of the AS-OCT is about 5μm,which seems to be a little difficult to evaluate and compare the three flap groups with different thicknesses, since each group only has a 10 μm difference in the thickness. In addition, boundaries between corneal flap and stroma became relatively ambiguous, especially in the central portion of the cornea 1 month after surgery. The examiner of the study was well trained and experienced who obtained all data carefully by himself in order to decrease the potential errors mentioned above. Previous studies usually examined the central flap thickness because this area is the thinnest part with the deepest ablation in LASIK [18–20]. Actually, the postoperative outcome of patients is determined by not only the central area but also the whole corneal flap, including paracentral and peripheral areas. Peripheral corneal distortions have been reported to relate with postoperative visual problems, such as decreased contrast sensitivity and poor night vision [21,22]. Therefore, in the present study, we measured the flap thickness at 30 locations in central, paracentral, and peripheral areas on 6 meridians of each eye by AS-OCT.

Femtosecond laser technology is not affected by corneal diameter, preoperative corneal thickness, oscillation frequency, and operation sequences on both eyes of the patient, whereas microkeratome LASIK is affected by these factors, making femtosecond laser-assisted LASIK superior in creating corneal flaps with accuracy and uniformity [23–27]. The corneal flap with a uniform thickness not only improves visual outcomes but also reduces complications, such as corneal ectasia, perforation, striae, and so on. The standard deviation (SD) of the thickness of the flaps made by IntraLaser femtosecond laser varied in previous studies. Li et al [28] reported that the SD of the flap thickness was ±9 μm (target 110 μm) and ±11 μm (target 120 μm). Zhou et al. [29] reported a SD of ±6.9 μm (target 100 μm), whereas Stahl et al [30] reported a SD of ±5.0 μm (target 110 μm). Prakash et al [12]reported much smaller SD of ±4.4 μm (target 100 μm), ±3.6 μm (target 110 μm), and ±4.0 μm (target 120 μm), respectively, which were similar to our results [±4.3 μm (target 100 μm), ±3.6 μm (target 110 μm), and ±2.9 μm (target 120 μm), respectively.

For each group, an ascending trend of the corneal flap thickness from the central area to the paracentral and peripheral areas was observed. Meanwhile, the deviation of the corneal flap thickness increased with the same trend: from the central area to the paracentral and peripheral areas. We also found that the deviation from the target whole flap thickness increased with this trend: from Group C to Group B and Group A. The uniformity of the corneal flaps was worse in Group A than in the other two groups, which may be explained in part by the systematical error of the laser platform. According to the formula for planarity index [12], the actual flap thickness is negatively related with the systematical error. In addition, the central cornea is biomechanically weaker than the peripheral cornea, and the posterior two-thirds of the corneal stroma are weaker than the anterior one-third [31–33]. A thinner flap occupies a larger part of the anterior one-third of the corneal stroma, resulting in stronger resistance and poorer planarity as compared with a thicker flap when making the flap with femtosecond laser. It is also difficult for femtosecond laser to ablate the same amount fibers between the peripheral and central areas of the cornea, explaining why the flap has a thick peripheral area and a thin central area instead of an absolute planar flap. All the measurement areas, however, keep a satisfactory regularity. Furthermore, we found that the thickness of the corneal flap made on a thinner cornea was slightly thinner than the one made on a thicker cornea, and vice versa. The explanation for this might be that the pressure on a thicker cornea was stronger than that on a thinner cornea, resulting in more compression on the thicker one.

The general principle of designing the target thickness of the flap is to make the flap as thin as possible under certain condition so as to preserve a thicker residual stroma, which is able to strengthen the corneal biomechanics [34] and reduce postoperative dry eye problems [35]. However, a flap with a thickness of < 100 μm may increase risks, such as free caps, buttonholes, flap slippage striae, haze, and so on [36–38]. On the contrary, many operators usually avoid making thick flaps because the thin residual stroma is probable associated with weak corneal biomechanics and iatrogenic corneal ectasia [34]. Taking a more balanced view, with the premise of enough residual stroma, a thick flap makes the procedure of operation easy and safe, and prevents epithelial implantation. Therefore, we must take into account the balance between enough residual stroma and operation convenience and safety when we design the flap thickness for patients with refractive error.

In our study, we used Intralaser FS60 femtosecond laser to create 100-μm, 110-μm, and 120-μm corneal flaps for patients with middle and low refractive error. The three groups all achieved satisfactory postoperative outcomes. To achieve maximum residual stroma and avoid flap complications, we recommend the following flap design strategy: select 100 μm as the target flap thickness while a patient’s preoperative spherical equivalent is at peak and the corneal thickness is at bottom; 120 μm can be considered while a patient’s preoperative spherical equivalent is at bottom and the corneal thickness is at peak; otherwise, 110 μm is a good choice while a patient’s preoperative assessment falls in between.

Generally speaking, it takes 3–6 months to normalize patients’ visual acuity, refractive status and the biological characteristics of cornea after LASIK surgery. Within this period, the visual and refractive outcomes are likely to change along with the change of flap perimeter and interface due to slow wound healing [39]. The complications, such as corneal ectasia, might not present until months or years after the surgery. Hence, longer term follow-up observation is needed to ascertain whether corneal ectasia is affected by different flap thicknesses.

In conclusion, our present study showed that the corneal flaps of three different thicknesses made by the Intralaser femtosecond laser were highly precise and predictable, yielding satisfactory visual and refractive outcomes. The strategy of creating customized corneal flaps according to different corneal thicknesses is a safe and effective way to correct low and moderate refractive error.
